# Supplementary material for: Whole-Transcriptome Analysis Identifies Gender Dimorphic Expressions of Mrnas and Non-Coding Rnas in Chinese Soft-Shell Turtle (Pelodiscus sinensis)
Source: Biology (Basel). 2022 May 29;11(6):834. doi: 10.3390/biology11060834 (PMC9219891; doi:10.3390/biology11060834)
Supplement: Supplementary file 1 [file biology-11-00834-s001.zip › biology-1721182-supplementary/Supplementary Files/The short curriculum vitae (CV) for all the authors.pdf]

Junxian Zhu,
a doctoral student at Shanghai Ocean University, focuses on sex determination and sex differentiation in *Pelodiscus sinensis*.
Luo Lei,
a doctoral student at Nanjing Agricultural University, focuses on sex determination and sex differentiation in *Pelodiscus sinensis*.
Chen Chen,
a staff with a PhD, works at the Pearl River Fisheries Research Institute, Chinese Academy of Fishery Sciences, focusing on the genetic breeding of *Pelodiscus sinensis*.
Yakun Wang,
a staff with a PhD, works at the Pearl River Fisheries Research Institute, Chinese Academy of Fishery Sciences, focusing on the reproduction and breeding of *Mauremys mutica* and *Macrobrachium rosenbergii*.
Xiaoli Liu,
a staff with a PhD, works at the Pearl River Fisheries Research Institute, Chinese Academy of Fishery Sciences, focusing on the reproduction and breeding of *Mauremys mutica*.
Lulu Geng,
a master's student at Nanjing Agricultural University, focuses on sex determination and sex differentiation in *Pelodiscus sinensis*.
Ruiyang Li,
a staff, works at the Pearl River Fisheries Research Institute, Chinese Academy of Fishery Sciences, focusing on the genetic breeding of *Pelodiscus sinensis* and *Mauremys mutica*.
Haigang Chen,
a staff, works at the Pearl River Fisheries Research Institute, Chinese Academy of Fishery Sciences, focusing on the genetic breeding of *Pelodiscus sinensis* and *Mauremys mutica*.
Xiaoyou Hong,
a staff with a PhD, works at the Pearl River Fisheries Research Institute, Chinese Academy of Fishery Sciences, focusing on conservation of genetic resources in *Pelochelys cantorii*.
Lingyun Yu,
an associate professor, works at the Pearl River Fisheries Research Institute, Chinese Academy of Fishery Sciences, focusing on the reproduction and breeding of *Macrobrachium rosenbergii*.
Chengqing Wei,
a staff, works at the Pearl River Fisheries Research Institute, Chinese Academy of Fishery Sciences, focusing on the genetic breeding of *Pelodiscus sinensis* and *Mauremys mutica*.
Wei Li,
an associate professor, works at the Pearl River Fisheries Research Institute, Chinese Academy of Fishery Sciences, focusing on sex determination and sex differentiation in *Pelodiscus sinensis*.

Xinping Zhu,
a professor, works at the Pearl River Fisheries Research Institute, Chinese Academy of Fishery Sciences, focusing on the reproductive, breeding, and conservation biology of *Pelodiscus sinensis*, *Mauremys mutica*, *Macrobrachium rosenbergii* and *Pelochelys cantorii*.
