# Supplementary figures and images for: Whole-Transcriptome Analysis Identifies Gender Dimorphic Expressions of Mrnas and Non-Coding Rnas in Chinese Soft-Shell Turtle (Pelodiscus sinensis)
Source: Biology (Basel). 2022 May 29;11(6):834. doi: 10.3390/biology11060834 (PMC9219891; doi:10.3390/biology11060834)

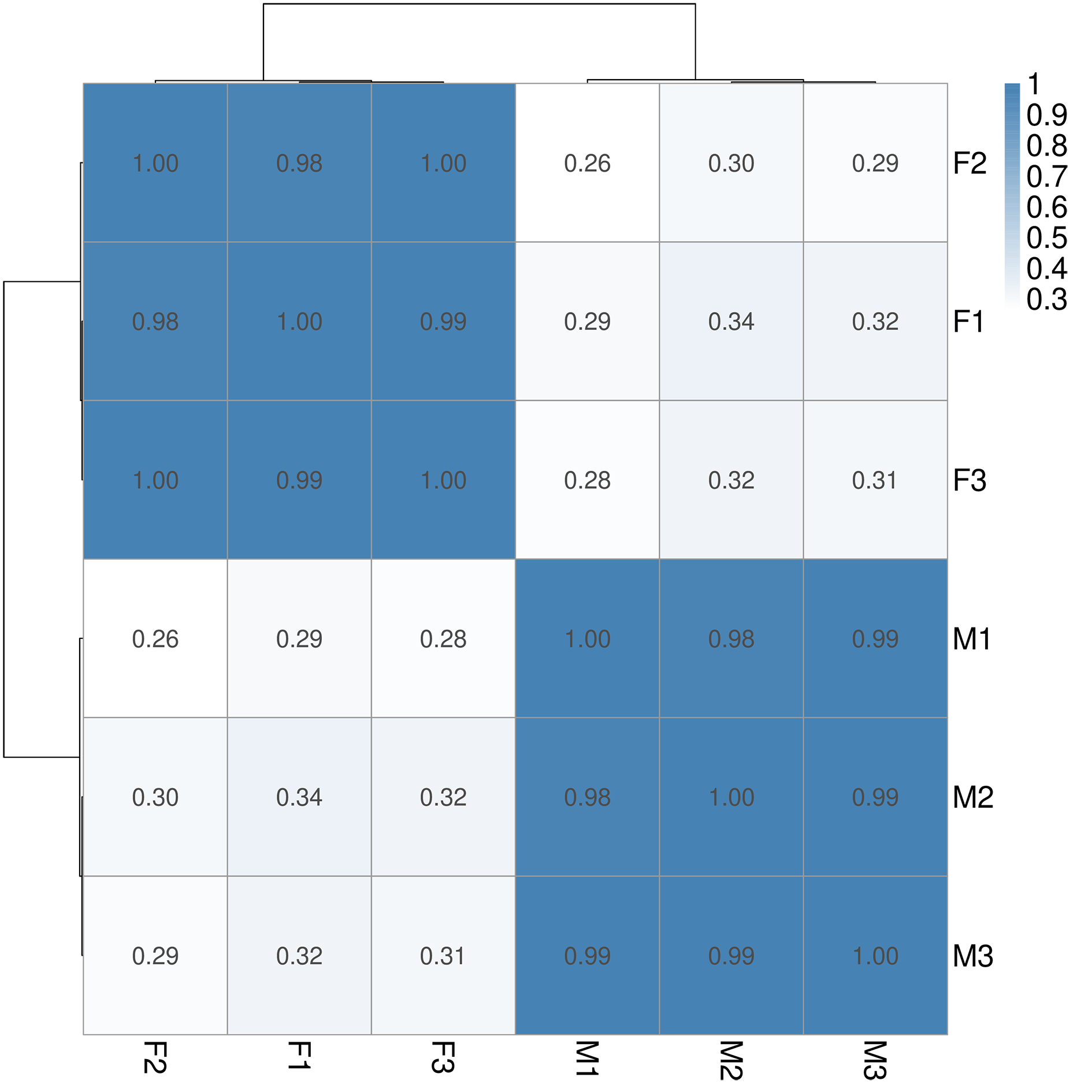

Supplement: Supplementary file 1 [file biology-11-00834-s001.zip › biology-1721182-supplementary/Supplementary Files/Figure S2. Pearson's correlation coefficient of each sample.tif]
